# Prevalence of Tuberculosis among migrants under national screening programs: a systematic review and meta-analysis

**DOI:** 10.1186/s41256-025-00424-y

**Published:** 2025-06-23

**Authors:** Qin Chen, Ningjun Ren, Shengya Liu, Zixin Qian, Mengdie Li, Aliyu Mustapha, Wei Luo, Jinghua Li, Wenxi Wang, Chun Hao

**Affiliations:** 1 Department of Medical Statistics, School of Public Health, Sun Yat-Sen University, Sun Yat-Sen University, Guangzhou, 510080 People’s Republic of China; 2 Sun Yat-Sen Global Health Institute, Institute of State Governance | Institute of International and Regional Studies, Sun Yat-Sen University, Guangzhou, 510275 People’s Republic of China; 3 Guangzhou International Travel Healthcare Center, Guangzhou, 510130 People’s Republic of China; 4 Shenzhen International Travel Health Care Center (Shenzhen Customs District Port Outpatient Clinics), Shenzhen Customs District, Shenzhen, 518033 People’s Republic of China; 5 Guangzhou Joint Research Center for Disease Surveillance, Early Warning, and Risk Assessment, Guangzhou, China; 6 The First Affiliated Hospital of Guangzhou Medical University, National Center for Respiratory Medicine, National Clinical Research Center for Respiratory Disease, State Key Laboratory of Respiratory Disease, Guangzhou Institute of Respiratory Health, Guangzhou, 510000 People’s Republic of China

**Keywords:** Tuberculosis screening, Migrants, Meta-analysis, Policy comparison

## Abstract

**Background:**

Tuberculosis (TB) continues to pose a significant global public health threat, particularly among migrant populations. Screening policies exist in many receiving countries but differ markedly, and there is limited pooled evidence on TB and latent TB infection (LTBI) prevalence among migrants under different screening frameworks. This systematic review and meta-analysis aims to synthesize TB and LTBI prevalence among migrants and compared national screening policies to inform evidence-based public health planning.

**Methods:**

PubMed, Embase, Web of Science and Cochrane Library were searched for studies published 2016–2023. Random-effects models generated pooled prevalence estimates with 95% CIs; subgroup analyses examined differences by screening stage, migrant category, and country-of-origin incidence. Sensitivity analyses tested robustness. Government and health-agency websites were systematically examined and scored to table national TB-screening requirements.

**Results:**

36 studies (26 TB, 21 LTBI) covering 40,738,331 migrants screened met inclusion criteria. The Pooled TB prevalence was 214.52/100,000 (95% CI 112.18–349.66) and LTBI prevalence 14.9% (95% CI 9.91–20.60). Countries employing both pre-entry screening and subsequent post-entry surveillance achieved the lowest TB prevalence (94.09/100,000). The highest burdens occurred among refugees/asylum seekers (439.25/100,000) and migrants from countries with TB incidence 300–499/100,000 (491.96/100,000). LTBI was most common when identified through post-entry screening (21.90%), those with multiple migrants (18.11%), and among migrants originating from countries with ≥ 500/100,000 TB incidence (30.90%). Policy comparison showed pre-entry screening is almost universal; the United States is the only country mandating systematic LTBI screening. Screening-scope scores were highest in traditional immigrant countries (16–20), intermediate in middle-income destinations such as China and Malaysia (10–14), and lowest in Nordic (4–8).

**Conclusions:**

This study emphasizes the importance of targeted TB screening, especially for migrants from high-prevalence regions and at-risk populations. Comprehensive pre- and post-entry TB screening, along with strengthened latent TB screening and surveillance for diverse migrant populations, is essential. Meanwhile enhanced collaboration to update screening policies are key to achieving the goal of TB eradication and provide practical insights for effective TB control.

**Supplementary Information:**

The online version contains supplementary material available at 10.1186/s41256-025-00424-y.

## Background

Tuberculosis poses a significant global public health challenge. According to the WHO Global Tuberculosis Report 2023, approximately one-quarter of the world's population carries a Mycobacterium tuberculosis infection, with most new cases originating from Southeast Asia and Africa [1]. In high-burden countries, TB cases are typically newly acquired, while in low-burden countries, they are often due to reactivation of latent TB infection (LTBI) among migrants [2]. The International Organization for Migration (IOM) estimates that in 2020, there were about 281 million migrants globally, accounting for 3.6% of the world’s population [3]. Migration plays a significant role in TB transmission, and reducing its associated risks is key to elimination efforts [4]. Effective TB screening, high-quality surveillance, and timely treatment among migrants are central to national and international policies aimed at disease control.

Over the past decade, many high-income countries have introduced TB control policies targeting migrant populations, including mandatory pre-entry screenings, and post-entry surveillance. Furthermore, unlike active TB, LTBI is more latent and insidious. Evidence suggests that 50% of TB cases among immigrants occur 2–5 years post-entry and are due to reactivation of LTBI [5, 6]. Despite the effectiveness of current global TB control strategies, the extensive reservoir of LTBI remains a major barrier to eliminating TB. Success will require significant reductions in both active TB and LTBI worldwide. In low-incidence countries, screening and preventive treatment of LTBI is an essential strategy for TB control, which can reduce the risk of subsequent morbidity in LTBI by 90% [7].

Currently, Several published meta-analyses on tuberculosis among migrants primarily focus on high-income countries that receive migrants from nations with high TB incidence rates or refugee populations, yet do not include middle-income countries [8–11], meanwhile, few meta-analysis have focused on LTBI among migrants, despite its significance in reactivation risk [12, 13], there is currently no consensus on the most effective screening strategies or policy frameworks [14–16]. At the same time, the heterogeneity of these policies across nations complicates the global management of TB, there is no internationally harmonized program for screening migrants for TB. Therefore, this systematic review and meta-analysis aims to synthesize the available evidence on TB and LTBI prevalence among migrants across different receiving countries and screening policies, and to provide a descriptive comparison of national TB screening policies, thereby supporting evidence-informed public health planning.

## Methods

### Study design

This systematic review and meta-analysis was registered on PROSPERO (registration No. CRD42024431263) and comprised two parts: the systematic review and meta-analysis, the policy description. The systematic review and meta-analysis followed the PRISMA (Preferred Reporting Items for Systematic Reviews and Meta-Analyses) checklist [17]. A completed PRISMA checklist for the review is presented in Appendix 1. The systematic review and meta-analysis section aimed to pool prevalence estimates of TB and LTBI among migrants based on eligible studies identified through a systematic literature search. In the policy comparison section, countries were selected based on the receiving countries identified in the included studies of the meta-analysis. Official TB screening policies were systematically retrieved, described, compared, and scored to reflect their relative levels of strictness and complexity.

### Systematic review and meta-analysis

**Search strategy.** Two authors independently conducted searches for studies published from January 1, 2016, to December 31, 2023, using PubMed, Embase, Web of Science, and Cochrane databases, with searches limited to the English language. Detailed search strategies are provided in Appendix 2. All articles were imported into the literature management software, Endnote. After removing duplicate studies, a three-tier screening process (title, abstract, and full-text) was performed. In cases of disagreements, independent evaluations by the two authors were reconciled through discussions with a third author.

**Eligibility criteria.** Inclusion criteria for this meta-analysis were studies that: (1) reported on the prevalence, incidence, or detection rates of TB, LTBI, or related terms; (2) involved participants undergoing entry screening processes for TB; and (3) were published within the designated study period from January 1, 2016, to December 31, 2023. Exclusion criteria included studies that: (1) did not focus on TB or its direct implications on public health screening; (2) lacked primary data or sufficient detail for extraction; and (3) were opinion pieces, editorials, or reviews without original data.

**Data collection.** Data from the included articles were extracted using Microsoft Excel templates tailored for academic literature. Each template featured columns addressing core research questions related to TB entry screening and broader article characteristics. The standardized form included fields for title, first author, publication year, study type, sample size, origin and receiving countries of migrants, study period, screening time point, screening method, migrant group, case count, and age. To ensure methodological consistency, pilot extractions were conducted on a select group of articles. Detailed templates are available in Appendix 3. Discrepancies during formal data extraction are referred for third-party review.

**Quality assessment**. According to the Standards for Reporting Observational Research in Epidemiology (STROBE), the study was evaluated for quality [18]. Appendix 4 gives a description of the 22 STROBE criteria designed to assess the quality of the studies included in our meta-analysis. Each checklist element was dichotomously scored: 1 point if the criterion was satisfactorily met and 0 points if it was either absent or inadequately addressed. Under this scheme, the maximum attainable score was 34, denoting full compliance with all STROBE reporting standards and, therefore, high-quality reporting. Two authors assessed the quality of the study, and in case of disagreement between the two authors, a third person arbitrated, and the conclusion was reached by combining the analysis results.

**Statistical analysis.** For statistical analysis, the prevalence of TB and LTBI were evaluated separately. The prevalence of TB and LTBI was combined in forest plots using Events and 95% confidence intervals (CI) as effect values. The prevalence of active TB was converted to the number of detections per 100,000 people, while LTBI prevalence was converted to the number of detections per 100 people. Heterogeneity was tested using Cochran' s *Q* test and I^2^ test, if there is a *Q* statistic of *p* < 0.05, or I^2^ > 50%, then there is significant inter-study heterogeneity, and meta-analysis was conducted using a random-effects model; if *p* ≥ 0.05 and I^2^ ≤ 50%, then heterogeneity is not significant, and a fixed-effects model was used. In addition, the publication bias estimates were assessed by observing the symmetry of funnel plots visually and statistically tested using Egger's test and Begg's Test, and sensitivity analysis assessed the outcomes' stability. Studies of the subgroups and meta-regression analyses were conducted to explore the sources of heterogeneity. The above statistical analyses were completed using R 4.2.3 software.

### Policy description

**Data sources and search strategy.** For the policy description, we systematically searched official government, health administration, the CDC and immigration-authority websites for TB entry-screening and visa medical-examination policies spanning all countries represented in the meta-analysis. Search terms for policy included 'TB screening policy,' 'TB screening requirements,' and 'TB screening guidelines,' with searches confined to English.

**Data extraction.** For each eligible policy we employed a standardized Microsoft Excel template to ensure complete and reproducible capture of all relevant variables. The standardized template includes fields for: receiving country, score, World Bank income level, migrant groups covered, TB screening method, TB screening time, LTBI screening method, LTBI screening time, etc. Detailed templates are available in Appendix 3. Discrepancies during formal data extraction are referred for third-party review.

**Policy scoring criteria.** We developed a scoring system to approximate the intensity of tuberculosis screening practices for migrants across countries. Each scored based on the comprehensiveness of their screening measures. The criteria are as follows: Duration of Stay: A threshold of “ ≥ 3 months” scores 4 points, while “ ≥ 6 months” scores 2 point; Screening by Countries of Origin: Screening applied to “ALL” countries scores 4 points, to “20–50” countries scores 2 points, to “ ≥ 100”, and other cases scoring 0 points; Migrant Categories: Screening of all migrant types scores 4 points, while screening only students or refugees scores 0 point; Screening Timing: Pre-entry screening scores 4 points, and post-entry screening scores 0 point; Differentiated Screening for LTBI: An additional 4 point is awarded if screening varies by age, otherwise 0 points. The sum of scores across these criteria provides a rough measure of the overall intensity of tuberculosis screening for migrants by country.

## Results

### Systematic review and meta-analysis

**Fig. 1:**
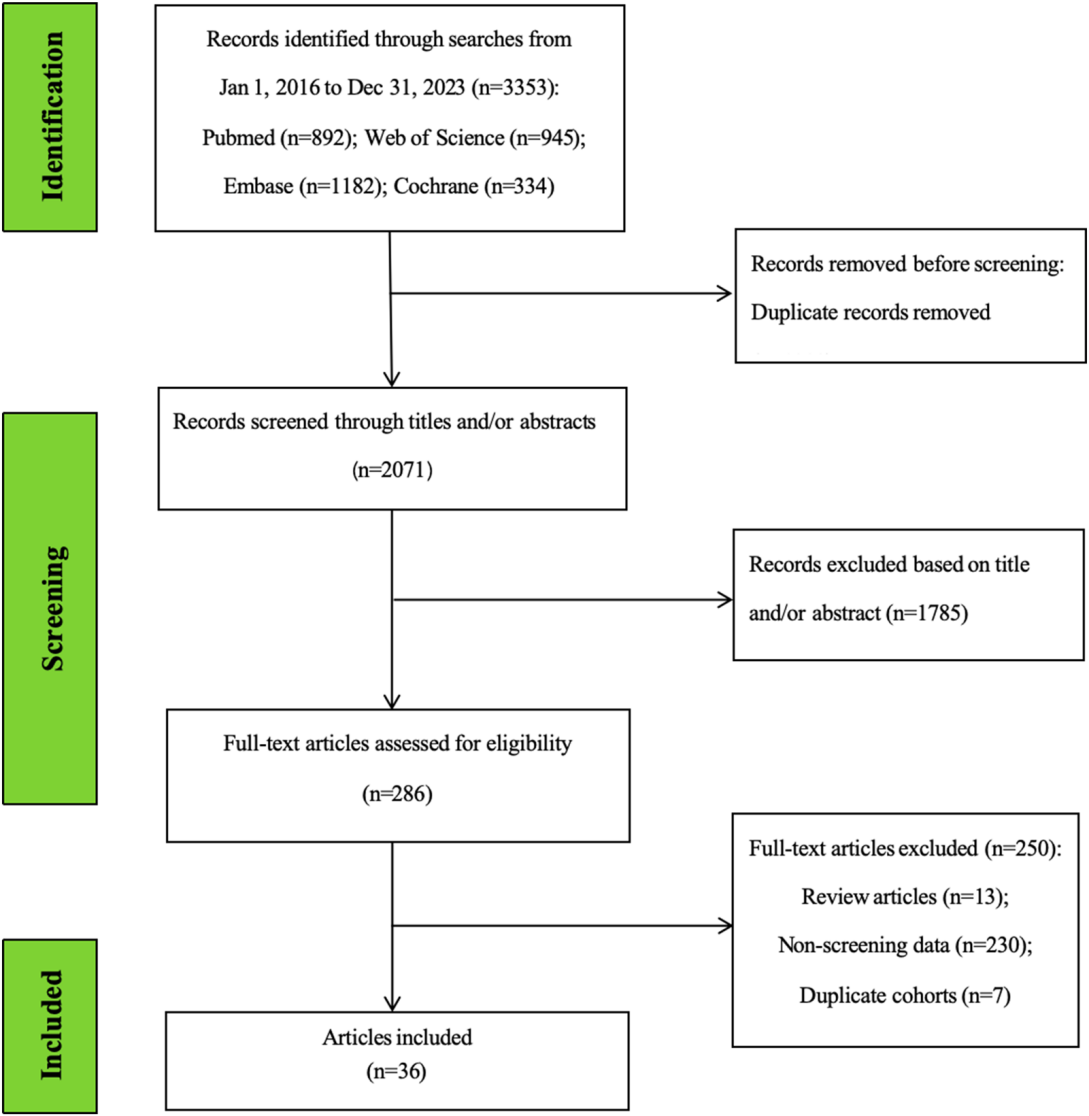
Flow diagram of the search and study selection

**Study selection. **We retrieved 3353 articles from four databases and removed 1282 duplicates. After title and abstract screening, 286 articles (8.5%) were retained. After full-text review, 250 articles were excluded. A final list of 36 (1.1%) articles were included for qualitative systematic review and quantitative meta-analysis (Fig. 1). Of these, 26 of the articles contained data on TB, and 21 had data on LTBI.

**Literature characteristics. **Table 1 presents the literature characteristics and quality assessment. The final sample of 36 studies included 40,738,331 migrants traveling to 17 countries: Australia, Canada, China, Denmark, Germany, Italy, Japan, Malaysia, Netherlands, New Zealand, Oman, Portugal, Singapore, South Korea, Sweden, United Kingdom, and United States. Most articles (78%) evaluated post-entry assessments, while 22% focused on pre-entry assessments. Study populations were diverse: 14 articles examined refugees or asylum seekers, four focused on labour or student visa holders, and the rest included mixed migrant groups. Chest X-ray was the most common diagnostic method (69%), followed by interferon-γ release assays (IGRA, 56%), tuberculin skin tests (TST, 36%), and sputum smear or culture (31%). Nearly half (47%) used two or more tests in parallel.

**Table 1:** Characteristics and STROBE ratings of the included migrant-TB screening studies

Author	Year	Sample	TB cases	LTBI cases	Study period	Screening time point	Screening methods	Migrants group	Receiving country	Country of origin	STROBE rating
Aldridge et al.[19]	2016	476,455	439	–	2005–2013	Post-entry	Sputum testing	All	United Kingdom	All	28
Alyaquobi et al. [20]	2020	1024	–	234	2018	Post-entry	IGRA	All	Oman	All	24
Båtshake et al.[21]	2023	7638	–	1424	2005–2018	Post-entry	QFT	All	Sweden,Netherlands,United Kingdom	All	20
Bennet et al.[22]	2017	2422	24	278	2015	Post-entry	IGRA,TST,CXR	Refugees/asylum seekers	Sweden	All	21
Berrocal-Almanza et al.[23]	2022	37,268	–	6640	2011–2018	Post-entry	IGRA	All	United Kingdom	All	22
Board et al.[24]	2016	9860	–	3249	2010–2013	Post-entry	TST,IGRA	Refugees/asylum seekers	United States	All	24
Bozorgmehr et al. [25]	2019	119,037	98	–	2002–2015	Post-entry	IGRA,TST,CXR	Refugees/asylum seekers	Germany	All	30
Bright et al.[26]	2020	9,840,960	3702	–	2015–2018	Post-entry	NA	All	Australia	All	29
Crawshaw et al. [27]	2018	9759	9	92	2013–2017	Pre-entry	CXR, sputum testing	Refugees/asylum seekers	United Kingdom	All	20
Dale et al. [28]	2020	14,671,064	3246	2,084,087	2006, 2011, 2016	Post-entry	NA	All	Australia	All	31
Douglas et al.[29]	2017	1,819,566	2887	–	2014	Pre-entry	CXR, sputum testing	All	Australia, Canada, New Zealand, UK, USA	All	29
Han et al. [30]	2019	18,494	33	–	2015–2017	Post-entry	CXR	Long-term visas, Work	China	Korea	25
Kawatsu et al.[31]	2018	391,160	333	–	2015	Post-entry	CXR, sputum testing	All	Japan	All	26
Kristensen et al. [32]	2019	142,314	1841	–	1993–2015	Post-entry	NA	Long-term visas	Denmark	All	30
Kumar et al.[33]	2021	78,062	69	8148	2014–2016	Post-entry	N/A	All	United States	All	28
Kumar et al.[34]	2020	6144	2	580	2014–2016	Post-entry	CXR, sputum test, TST,IGRA	Refugees/asylum seekers	United States	Iraq, Afghanistan	28
Kumar et al.[35]	2020	19,167	156	159	2009–2017	Pre-entry	CXR, sputum test, TST,IGRA	Refugees/asylum seekers	United States	Iraq, Afghanistan	23
Lim et al. [36]	2021	3584	–	727	2016–2019	Post-entry	IGRA	Work	Singapore	All	26
Liu et al. [37]	2020	2,102,415	4225	30,574	2013–2016	Pre-entry	TST, IGRA, CXR, sputum testing	All	United States	All	27
Lowenthal et al. [38]	2016	12,544	8	4237	2008–2013	Post-entry	IGRA, TST, CXR, sputum testing	All	United States	All	29
Menezes et al.[39]	2022	2,302,260	1658	–	2015–2018	Post-entry	IGRA,TST,CXR	All	Italy	All	25
Nederby Öhd et al. [40]	2021	5470	–	1364	2015–2018	Post-entry	IGRA	Refugees/asylum seekers	Sweden	All	24
Paulino et al.[41]	2016	2,193,594	376	–	2008–2012	Post-entry	NA	All	Portugal	All	26
Prestileo et al.[42]	2021	3710	61	501	2014–2017	Post-entry	IGRA,TST,CXR	Refugees/asylum seekers	Italy	All	28
Severi et al.[43]	2016	200,199	350	–	2009–2010	Post-entry	CXR	All	United Kingdom	All	23
Stadtmüller et al. [44]	2021	1,102,918	2093	–	2014–2016	Post-entry	CXR	Refugees/asylum seekers	Germany	All	25
Stærke et al.[45]	2022	1507	–	189	2014–2020	Post-entry	IGRA	Refugees/asylum seekers	Denmark	All	31
Taylor et al.[46]	2016	67,334	–	8231	2010	Pre-entry	TST,IGRA	All	United States	All	29
Toms et al. [47]	2017	2,554,230	887	–	2014	Post-entry	NA	All	Australia	All	25
Trauer et al.[48]	2021	2,381,217	1263	–	2014–2017	Pre-entry	CXR	All	Australia	All	28
Urban et al.[49]	2022	70,290	759	–	1993–2019	Pre-entry	CXR	Refugees/asylum seekers	United States	All	25
Usdin et al.[50]	2017	440	2	71	2014	Post-entry	Questionnaire, IGRA	Students	United Kingdom	Countries with incidence > 40/100,000	22
Wien et al. [51]	2020	15,729	3	13	2009–2017	Pre-entry	CXR, sputum test, TST,IGRA	Refugees/asylum seekers	United States	Iraq, Afghanistan	27
Wong et al.[52]	2020	430	–	55	2018–2019	Post-entry	TST/IGRA/CXR	Refugees/asylum seekers	Malaysia	All	24
Yu et al. [53]	2021	8108	–	2275	N/A	Post-entry	QFT	All	South Korea	All	32
Yun et al. [54]	2016	8148	–	1560	2006–2012	Post-entry	TST,IGRA	Refugees/asylum seekers	United States	All	26

**Quality assessment.** Among the 36 observational studies assessed using the adapted STROBE checklist, total scores ranged from 20 to 32, the average score is 26. When standardized to a maximum of 34 points, the average reporting completeness was 74.5%. Item-by-item analysis revealed that justification of discussion of the generalizability (reported in 30.5% of studies), explanation of missing data (22.2%), flow diagram (16.6%), and sensitivity analyses (13.8%) were the most frequently underreported elements. In contrast, research objectives, study aims, and basic statistical methods were reported in over 90% of the studies. 16 (44.4%) of the 36 studies as high quality, 20 (55.6%) as moderate. Overall, the included studies were of moderate to high quality. Details are provided in Appendix 5.

**Fig. 2:**
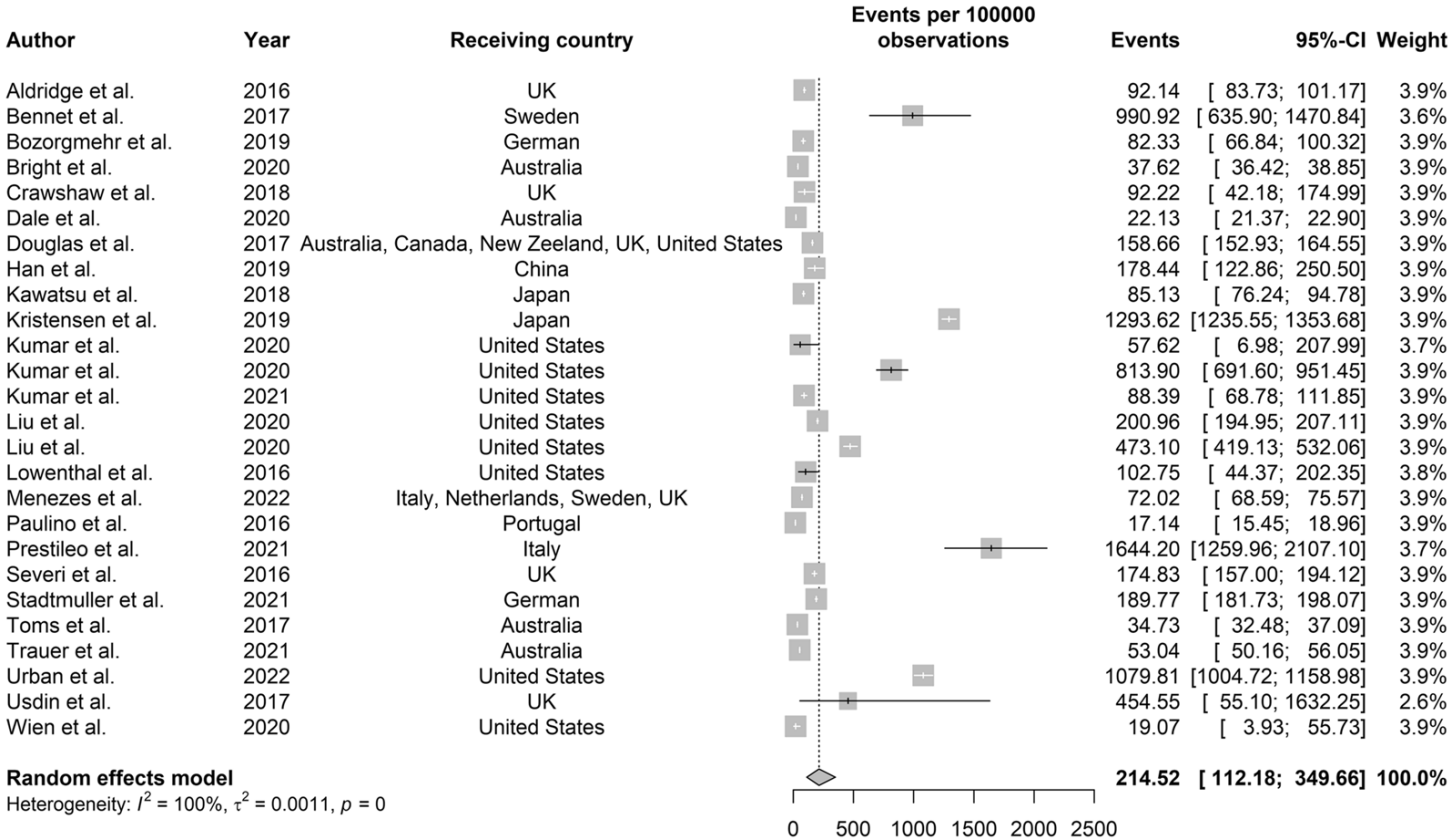
Forest plot of cumulative prevalence of TB

**TB screening for migrants.** Figure 2 shows TB detection among 40,585,259 migrants from 26 studies with estimated TB prevalence ranging from 17.14 to 1644.20 (per 100,000). The pooled estimate of cumulative prevalence of TB among immigrants was 214.52 per 100,000 (95% CI: 112.18–349.66).

**Fig. 3:**
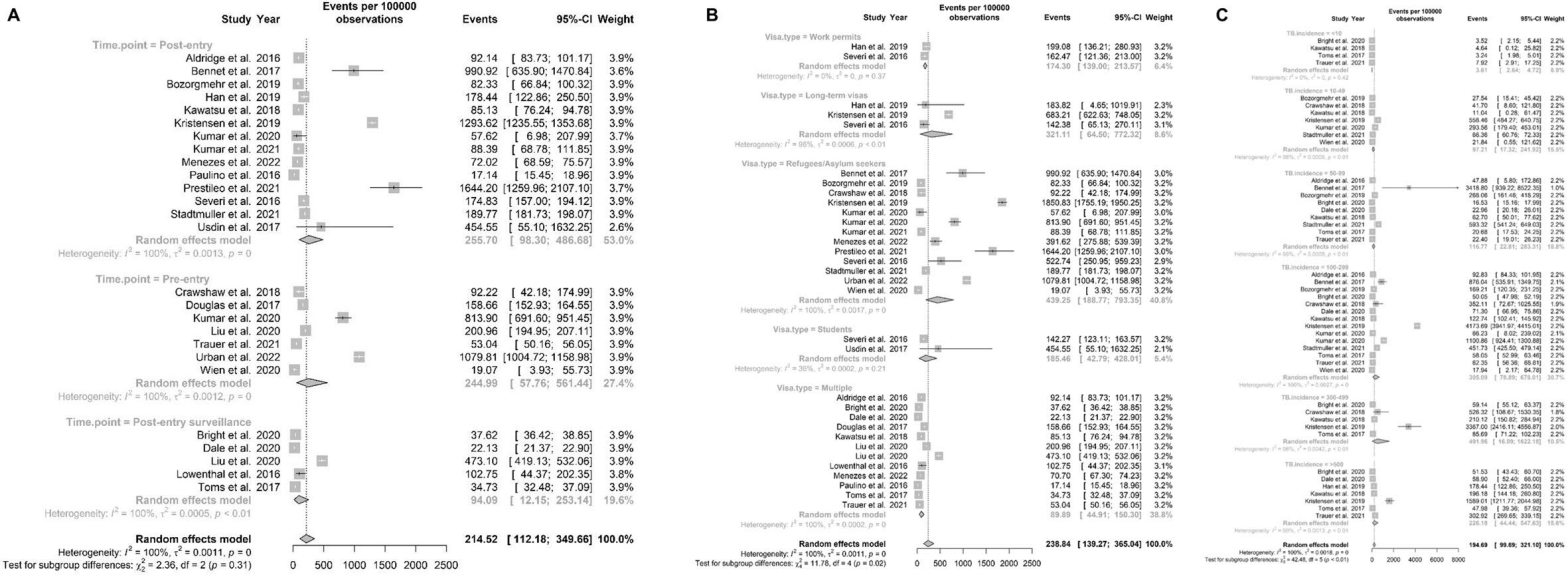
Forest plots of cumulative prevalence of TB, stratified by screening time point, migrant group, WHO-estimated TB incidence in country of origin

**Subgroup analysis of TB.** These were performed by screening time points, visa types, and prevalence by WHO-estimated country of origin. The pooled estimate of the prevalence was 255.70 per 100,000 (95% CI: 98.30–486.68) in 14 studies of post-entry screening, 94.09 per 100,000 (95% CI: 12.15–253.14) in 5 studies of post-entry surveillance, and 244.99 per 100,000 (95% CI: 57.76–561.44) in 7 studies of pre-entry screening. Figure 3A shows that screening time point, the pooled estimates of cumulative prevalence differed significantly between subgroups. Figure 3B shows that the pooled estimate of the prevalence in the subgroups of refugees/asylum seekers, long-term visas, students, work permits and multiple immigrants visas were 439.25 (95% CI: 188.77, 793.35), 321.11 (95% CI: 64.50, 772.32), 185.46 (95% CI:42.79, 428.01), 174.30 (95% CI:139.00, 213.57), 89.89 (95% CI: 44.91, 150.30), respectively. Of these, the refugee/asylum seekers subgroup had the highest prevalence. Figure 3C presents pooled TB prevalence estimates by WHO-estimated country of origin incidence in six subgroups: < 10, 10–49, 50–99, 100–299, 300–499, ≥ 500, the pooled estimate of the prevalence were 3.61 (95% CI: 2.64, 4.72), 94.21 (95% CI: 17.32, 241.92), 116.77 (95% CI: 22.81, 283.31), 305.09 (95% CI: 78.89, 678.01), 491.96 (95% CI: 16.09, 1622.18), 226.18 (95% CI: 44.44, 547.63), respectively. The "300–499" subgroup had the highest prevalence. Notably, for immigrants from countries with TB incidence rates of 10–299, their prevalence in the host country often exceeded the baseline rates. In contrast, for those from countries with TB incidence > 500, observed prevalence among immigrants was generally lower than in their countries of origin.

**Fig. 4:**
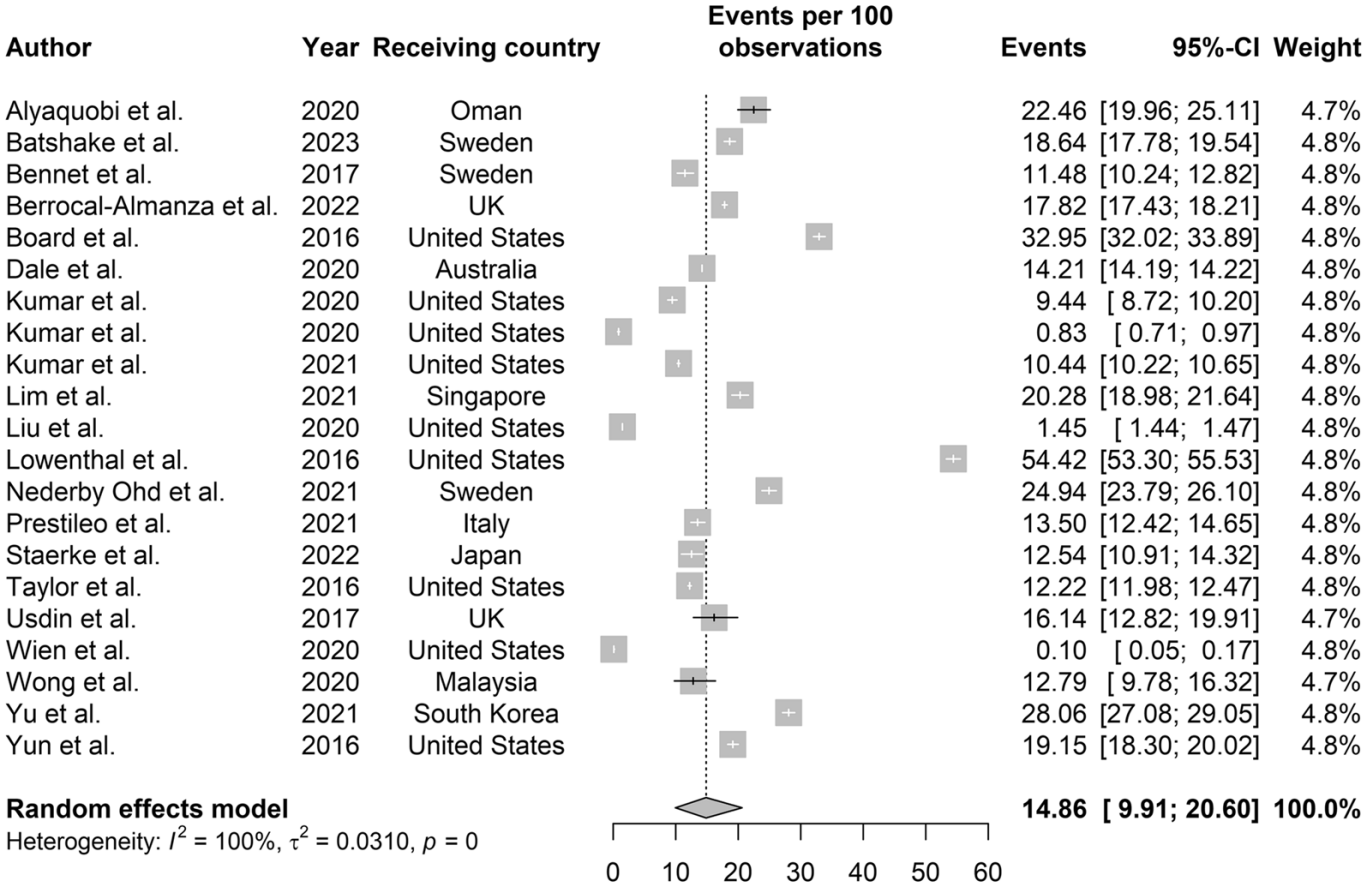
Forest plot of cumulative prevalence of LTBI

**LTBI screening for migrants.** Figure 4 shows LTBI detection among 17,054,473 migrants from 21 studies with an estimated LTBI prevalence of 14.86% (95% CI: 9.91, 20.60).

**Fig. 5:**
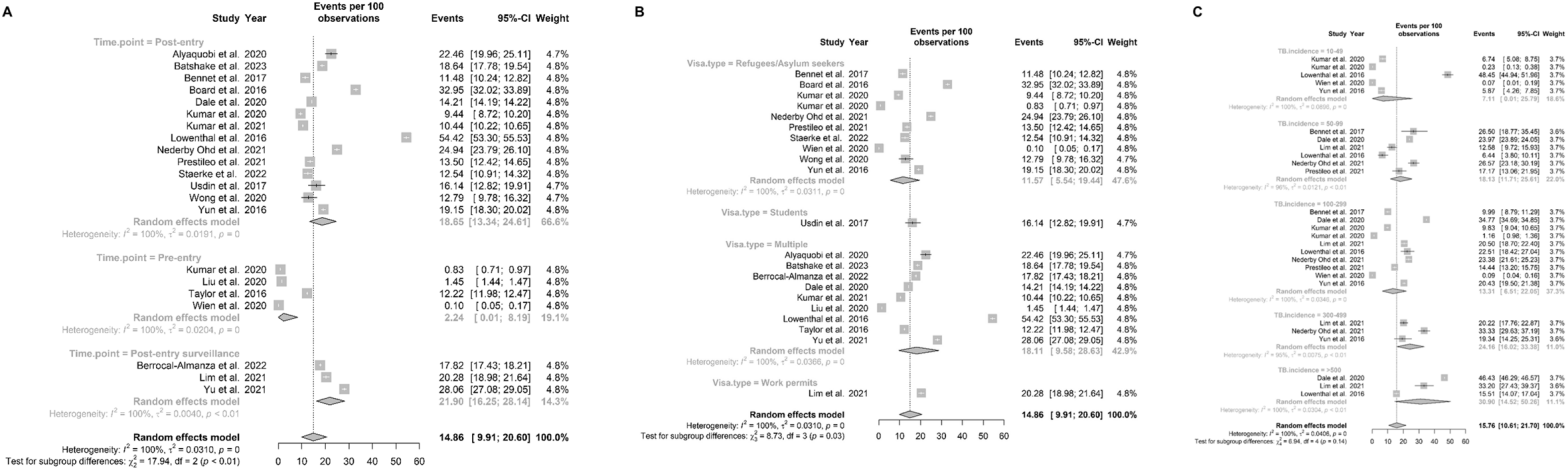
Forest plots of cumulative prevalence of LTBI, stratified by screening time point, migrant group, WHO-estimated TB incidence in country of origin

**Subgroup analyses of LTBI. **Figure 5A shows that the screening time point of LTBI showed that pooled estimates of cumulative prevalence differed significantly between subgroups. The pooled estimate of the prevalence was 18.65% (95% CI: 13.34–24.61) in 14 studies of post-entry screening, 21.90% (95% CI: 16.25–28.14) in 3 studies of post-entry surveillance and 2.24% (95% CI: 0.01–8.19) in 4 studies of pre-entry screening. Figure 5B shows that LTBI subgroup analyses by the immigrant group found the highest prevalence among multiple immigrants (18.11%, 95% CI:9.58, 28.63) and the lowest prevalence among refugees/asylum-seekers (11.57%, 95% CI:5.54, 19.44); and Fig. 5C shows that subgroups of the WHO-estimated country-of-origin TB incidence at the " > 500" level had the highest prevalence (30.90%, 95% CI: 14.52%, 50.26%).

**Fig. 6:**
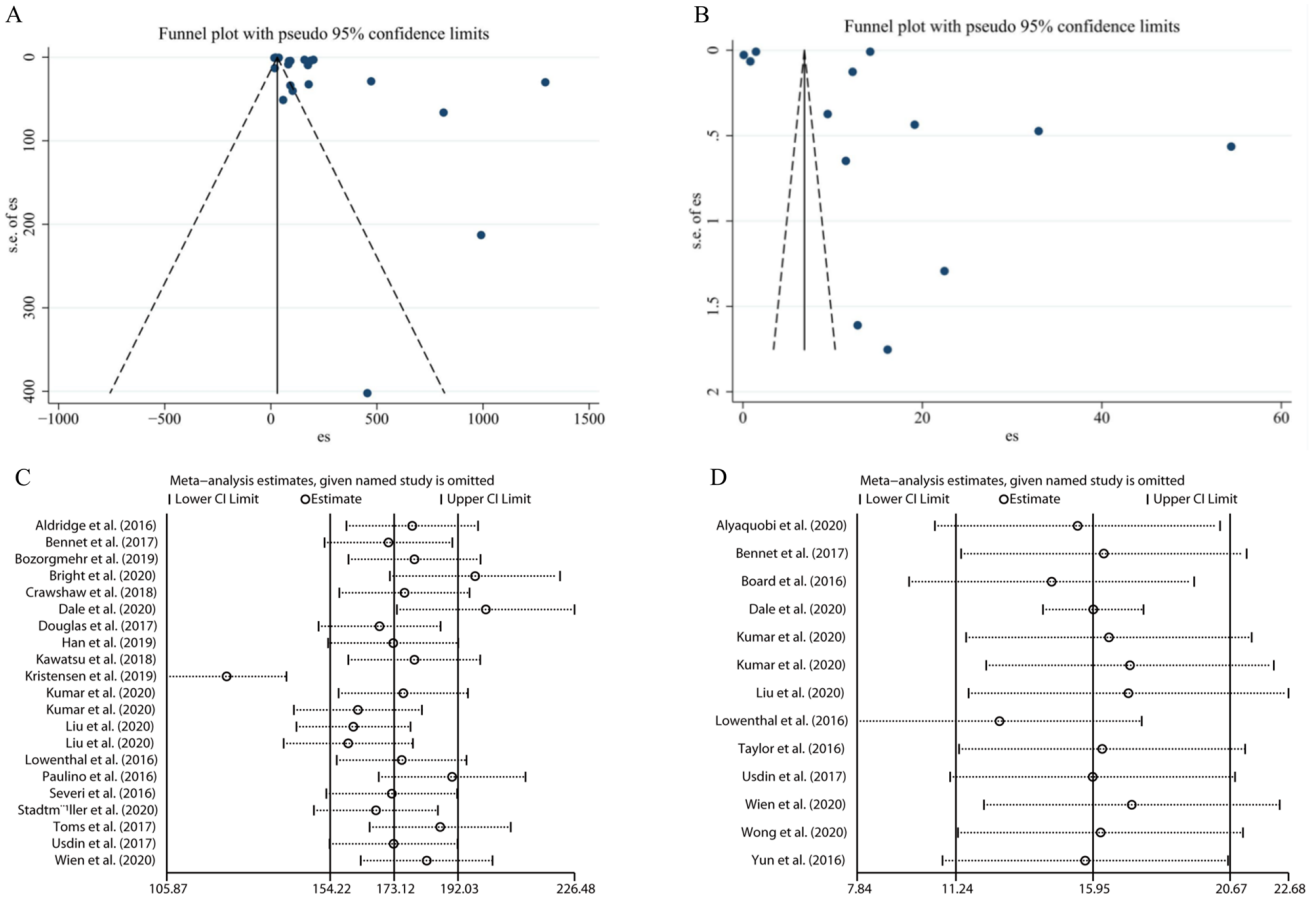
Publication bias funnel and sensitivity analysis for TB and LTBI

**Heterogeneity and Publication Bias. **Substantial heterogeneity was observed among the included studies for both TB and LTBI prevalence estimates (*I*^*2*^ = 100%), which may be attributed to differences in study populations, screening approaches, and host country contexts. Publication bias was assessed using funnel plots along with Begg’s and Egger’s tests, the distribution of points in both funnel plots appeared asymmetric. As shown in Fig. 6A, for TB studies, Begg’s test yielded *p* = 0.381 while Egger’s test showed statistical significance (*p* = 0.003), indicating potential publication bias, likely influenced by small-sample studies. As shown in Fig. 6B, for LTBI studies, Begg’s and Egger’s tests produced *p*-values of 0.669 and 0.950 respectively, suggesting no significant evidence of publication bias.

**Sensitivity analysis. **This was conducted using a one-by-one exclusion method to evaluate the impact of individual studies on the combined results. Figure 6C shows that the prevalence of TB varied from 185.38 per 100,000 (95% CI: 98.73, 229.04) to 227.34 (95% CI: 119.76, 369.00). The stability of the combined results was favorable. Figure 6D shows that the combined results for LTBI were also more stable with a range of variation from 13.34% (95% CI: 9.22, 18.09) to 16.18% (95% CI: 11.38, 21.63).

### Policy description

A total of 17 countries were included in this study, all of which are high-income nations except for Malaysia and China. The screening policy search strategy involved reviewing government websites, CDC platforms, and official health department sites for each country. This analysis identified both similarities and differences in TB screening policies for migrants, revealing some variance in requirements and procedures. Notably, all countries, except for a few European nations, restrict the long-term stay of migrants diagnosed with tuberculosis. Among the 17 countries, entry screening for tuberculosis is mandatory in 16, with the exception of Italy, where screening is voluntary.

Table 2 shows pronounced variation in migrant TB screening policies, summarized by composite scores which were assigned based on five criteria: duration of stay required for screening, breadth of countries of origin targeted, inclusivity of migrant categories, timing of screening (pre- or post-entry), and whether included LTBI testing.

**Table 2:** Country-level migrant tuberculosis entry-screening frameworks: composite policy-intensity scores for receiving nations

Receiving Countries	Score	Income Level by World Bank	Incidence WHO estimated	Duration of Stay	Countries of Origin	Migrants Groups	Age	Active TBScreening Methods	Active TBScreening Time	LTBI Screening Methods	LTBI Screening Time
United States [55]	20	High-income	2.6	≥ 3 months	All	All	≥ 15	Chest X-ray	Pre-entry		
					≥ 20		≥ 12	IGRA	Pre-entry	IGRA	Pre-entry
Oman [56]	18	High-income	5.9	≥ 3 months	≥ 50	All	≥ 18	Chest X-ray	Pre-entry	IGRA	Pre-entry
Australia [57]	16	High-income	6.5	≥ 3 months	All	All	≥ 11	Chest X-ray	Pre-entry		
					≥ 50		2–10	IGRA/TST	Pre-entry		
Canada [58]	16	High-income	5.3	≥ 6 months	≥ 30	All	≥ 11	Chest X-ray	Pre-entry	Health Surveillance	Post-entry
UK [59]	16	High-income	6.3	≥ 6 months	≥ 40	All	≥ 11	Chest X-ray	Pre-entry		
					≥ 150		16–35	Chest X-ray	Pre-entry	IGRA	Post-entry
South Korea [60]	14	High-income	39	≥ 3 months	≥ 50	All	All	Chest X-ray	Pre-entry		
Singapore [61]	14	High-income	45	≥ 6 months	All	All	All	Chest X-ray	Pre-entry		
New Zealand [62]	12	High-income	6.8	≥ 6 months	≥ 40	All	≥ 11	Chest X-ray	Pre-entry		
China [63]	10	Middle-income	55	≥ 6 months	All	All	All	Chest X-ray	Post-entry		
Malaysia [64]	10	Middle-income	97	≥ 6 months	All	Student visa, Work permit	All	Chest X-ray	Pre-entry		
German [65]	8	High-income	5	≥ 3 months	All	Refugees/Asylum seekers	≥ 15	Chest X-ray	Post-entry		
Japan [66]	8	High-income	11	≥ 3 months	China, Indonesia, Myanmar, Nepal, Philippines, VietNam	Student visa, Work permit	All	Chest X-ray	Pre-entry		
Netherlands [67]	8	High-income	4.1	≥ 3 months	≥ 100	All	≥ 18	Chest X-ray	Post-entry		
						All	< 18	TST	Post-entry		
Portugal [68]	8	High-income	16	≥ 3 months	All	Refugees/Asylum seekers	≥ 15	Chest X-ray	Post-entry		
Denmark [69]	6	High-income	3.8	≥ 3 months	≥ 100	Refugees/Asylum seekers	≥ 18	Chest X-ray	Post-entry		
Sweden [70]	4	High-income	3.8	≥ 3 months	≥ 100	Refugees/Asylum seekers	All	Chest X-ray	Post-entry		

Scores across countries ranged from 4 to 20, indicating considerable variation in screening intensity. High-scoring countries (20 and 18), the United States and Oman, implement more comprehensive screening, applying to all migrants from all countries of origin, with lower duration of stay thresholds, pre-entry timing, and age differentiated LTBI screening. In contrast, lower-scoring countries (4–8), including Germany, Denmark, Sweden, Japan, Portugal, and the Netherlands, tend to restrict screening to specific migrant groups (such as refugees or students), or rely primarily on post-entry surveillance without LTBI component, which reflects a resource-prioritization model and flexible public health strategy. Countries with intermediate scores (10–16), such as Australia, Canada, the UK, South Korea, Singapore, New Zealand, China, and Malaysia, generally require TB screening for longer-term migrants, often applying pre-entry screening for stays of three or six months or more, but typically do not mandate LTBI screening. Notably, China and Malaysia have relatively lower scores within this group: China implements universal post-entry screening, while Malaysia restricts pre-entry screening to applicants from high-burden countries. Countries with higher scores are typically traditional immigrant countries with diverse migrant categories, and middle-income countries with medium scores, while those with lower scores, such as Nordic countries primarily receive refugees and asylum seekers.

## Discussion

To our best knowledge, no prior meta-analysis has comprehensively assessed TB and LTBI prevalence among migrants across both high- and middle-income receiving countries. It demonstrates a high prevalence of both TB and LTBI among migrant populations, with substantial variability based on screening type, visa type, and country of origin. The overall TB prevalence of 214.52 per 100,000 and LTBI prevalence of 14.86% align with WHO estimates, suggesting our findings provide a reliable benchmark for understanding TB risk among migrants. Notably, the highest prevalence of TB was found in asylum seekers and refugees, and migrants from high-incidence countries, these results underscore the importance of tailored TB control measures for high-risk migrant subgroups. Meanwhile, post-entry screening groups exhibited higher LTBI prevalence compared to pre-entry screened groups, underscoring the need for comprehensive TB control strategies that include ongoing surveillance and follow-up screening. The results of the policy comparison emphasize the need for improved entry screening, continuous post-entry surveillance, and enhanced long-term TB screening and treatment to effectively manage the risk of imported TB.

The overall TB prevalence of 214.52 per 100,000 is lower than previous meta-analysis. This discrepancy could be attributed to differences in study populations, geographical regions, and time periods. Many previous meta-analysis have focused on specific populations or high-risk regions, which may explain the higher prevalence rates observed in those studies. For example, Greenaway et al. reported a pooled prevalence of 350 per 100,000 among migrants screened across EU/EEA countries [71]. Bozorgmehr et al. found an even higher prevalence of 347 per 100,000 in asylum seekers screened upon arrival in Germany [9]. Similarly, Aldridge et al. identified a pooled TB prevalence of 336 per 100,000 in migrants undergoing pre-entry screening in low-incidence countries [11]. These studies often concentrated on refugees, asylum seekers, or migrants from high TB-burden countries. In contrast, our study included a broader and more diverse migrant population, encompassing a variety of visa types, screening modalities, and both high- and middle-income destination countries, thereby offering a more comprehensive understanding of TB prevalence across different migration contexts.

Our subgroup analysis of TB identified specific high-risk groups based on screening timing and visa categories. By comparing pre-entry and post-entry screening, we found that the prevalence of TB was significantly lower among those who completed pre-entry screening and were also monitored post-entry compared to post-entry screening. This suggests that pre-entry screening is more effective in identifying individuals who are at risk of TB before they enter the country, allowing for earlier detection and intervention. The advantage of pre-entry screening lies in its ability to identify latent infections that may otherwise go undetected until later stages of migration or settlement. To our knowledge, no previous meta-analyses have systematically compared TB prevalence across different screening time points among migrant. o our knowledge, no previous meta-analyses have systematically compared TB prevalence across different screening time points among migrant. Moreover, our analysis by visa types revealed that certain groups, such as workers or refugees, showed higher TB prevalence rates. These populations might benefit particularly from pre-entry screening to ensure that they are not carrying undiagnosed infections when they arrive. This emphasizes the need for policy adjustments that prioritize pre-entry TB screening for high-risk visa categories to reduce the burden of TB in the long term. Studies by Liu [72], Lönnroth [73], and Chan [74] highlight various components of TB control strategies for migrants, underscoring the significance of pre-migration TB screening, ensuring universal access to quality care throughout the migration process, and implementing post-entry interventions for migrants identified with abnormal findings during pre-entry screening.

The subgroup analysis provided valuable insights into LTBI prevalence among different populations. Post-entry screening revealed a significantly higher LTBI prevalence (18.65%) compared to pre-entry screening (2.24%), suggesting the effectiveness of post-entry screenings in identifying LTBI. Analysis by entrant type showed varying LTBI prevalence, likely influenced by living and working conditions, as well as the overall health status of the entrants. Furthermore, considering the WHO-estimated incidence in the country of origin, individuals from high TB incidence countries (" > 500" subgroup) had the highest LTBI prevalence (30.90%), emphasizing the necessity of heightened screening and preventive measures for these high-risk regions. However, notable discrepancies were observed in certain subgroups ("10–49" and "100–299"), with higher LTBI prevalence compared to the WHO estimates, while others ("300–499" and " > 500") showed lower rates. These deviations have also been noted in previous studies, indicating that inconsistency between observed prevalence and WHO incidence data is not unique to this review [15]. Several possible explanations exist. One is the "healthy migrant effect", in which individuals who are able to migrate are typically healthier than the general population in their country of origin, thereby potentially underrepresenting the true disease burden [9]. Additionally, variations in screening methodology, including differences in diagnostic tests (TST or IGRA) [13], screening time points (pre-entry or post-entry), and the implementation of universal versus risk-based screening protocols [15], could all contribute to heterogeneity. Some studies may have missed recent infections or failed to capture LTBI reactivation occurring beyond initial screening periods [75]. Finally, data limitations, such as small sample sizes, wide confidence intervals, and uneven regional representation, particularly for middle- and low-income receiving countries, may also explain some of the observed variation. These factors highlight the complexity of interpreting LTBI prevalence in migrant populations and the need for standardized, longitudinal research to validate findings across different contexts.

In reviewing the cross-national comparison of tuberculosis screening policies, it is notable that countries with more stringent entry requirements, such as the United States, Canada, Australia, and New Zealand, are traditionally immigrant receiving nations. These countries have long standing immigration infrastructures and public health systems that prioritize early disease detection to minimize health-related integration challenges and protect public health security [29]. Their TB screening policies are often embedded within broader immigration health frameworks, making pre-entry screening and post-entry follow-up routine components of the visa process. By contrast, many Nordic countries such as Sweden, Denmark, and Norway adopt relatively more lenient or selective screening policies [39, 76]. This approach may reflect both different migration patterns and public health philosophies. Nordic countries typically accept a higher proportion of humanitarian migrants and refugees, and their health systems emphasize universal access and equity. In such contexts, selective post-entry screening, targeting high-risk groups rather than implementing universal screening, may be seen as more ethically justified and resource-efficient [15, 25, 71]. Moreover, these countries often integrate TB control into broader migrant health assessments conducted after arrival, rather than isolating it as a separate immigration criterion. This variation highlights how screening policy stringency is not solely determined by TB burden or epidemiological data, but also shaped by broader migration histories, public health values, and administrative capacities [4].

This study's primary strength lies in its comprehensive inclusion of immigrant populations from countries with varying levels of TB risk. In contrast to the majority of previous meta-analysis, which have concentrated on low-risk receiving countries and high-risk countries of origin [9, 19], this study encompassed both high- and low-risk receiving countries and countries of origin, thereby offering a more comprehensive perspective on the dynamics of TB. Meanwhile, few meta-analyses have explicitly examined LTBI prevalence in the context of immigrant entry screening, despite its importance for future TB risk. By addressing this gap and combining epidemiological data with cross-country policy comparisons, the analysis reveals variations in screening strictness and provides actionable insights for optimizing TB control strategies.

This study has several limitations, primarily the high heterogeneity among included studies and the inherent constraints of meta-analysis, similar to previous prevalence research [9, 19, 22, 26]. Additionally, data coverage was uneven, with limited information from middle- and low-income receiving countries which is similar to Aldridge et al. meta-analysis [11]. Finally, the policy comparison provides only a descriptive overview rather than a formal evaluation of effectiveness, and our exploratory scoring system, while useful for broad comparison, is subjective and does not apply differential weighting across domains. Nevertheless, such approaches are common in comparative health policy research and offer valuable initial insights into cross-country variations in TB screening practices. such approaches are common in comparative health policy research [77] and offer valuable initial insights into cross-country variations in TB screening practices.

## Conclusions

The findings of this study emphasize the importance of TB and LTBI screening among migrants while highlighting significant differences in screening policies across countries. Despite inherent limitations such as heterogeneity and reliance on cross-sectional data, the findings provide strong evidence for the need to refine TB screening strategies. Enhancing entry and post-entry screening, addressing psychosocial barriers, and utilizing emerging technologies are critical steps in reducing the global burden of TB among mobile populations. Further longitudinal research is essential to validate these findings and guide future policy reforms aimed at reducing the global TB burden among mobile populations. The findings also suggest potential directions for future efforts, including standardizing screening protocols, improving LTBI follow-up and treatment adherence, and strengthening coordination across national policies to better address the needs of high-risk migrant populations.

## Supplementary Information

Additional file 1.

Additional file 2.

Additional file 3.

Additional file 4.

Additional file 5.

## Data Availability

The data used in this study were gathered from publicly available studies.

## Abbreviations

TB: Tuberculosis
LTBI: Latent tuberculosis infection
WHO: World Health Organization
IOM: International Organization for Migration
TST: Tuberculin Skin Test
IGRA: Interferon-γ Release Assays
CDC: The Centers for Disease Control and Prevention
STROBE: Standards for Reporting Observational Research in Epidemiology
PRISMA: Preferred Reporting Items for Systematic Reviews and Meta-Analyses
CI: Confidence Intervals
EU/EEA: European Union/European Economic Area
