# Supplementary material for: Prevalence of Tuberculosis among migrants under national screening programs: a systematic review and meta-analysis
Source: Glob Health Res Policy. 2025 Jun 23;10:24. doi: 10.1186/s41256-025-00424-y (PMC12183900; doi:10.1186/s41256-025-00424-y)
Supplement: Supplementary file 2 — Additional file 2. [file 41256_2025_424_MOESM2_ESM.docx]

**PubMed**

((((((tuberculosis[MeSH Terms]) OR active tuberculosis[MeSH Terms]) OR LTBI[MeSH Terms]) OR latent tuberculosis infection[MeSH Terms])) AND (((((prevalence) OR incidence) OR detection rate) OR epidemiology) OR screening)) AND ((((((migrant) OR immigrant) OR foreigner) OR alien) OR travel) OR visa)

**Embase**

#1. prevalence OR incidence OR (detection AND rate) OR epidemiology OR screening

#2. migrant OR immigrant OR foreigner OR alien OR travel OR visa

#3. tuberculosis OR (active AND tuberculosis) OR ltbi OR (latent AND tuberculosis AND infection)

#4. #1 AND #2 AND #3

**Cochrane**

#1 (tuberculosis) OR (active tuberculosis) OR (LTBI) OR (latent tuberculosis infection) (Word variations have been searched)

#2 (prevalence) OR (incidence) OR (detection rate) OR (epidemiology) OR (screening) (Word variations have been searched)

#3 (migrant) OR (immigrant) OR (foreigner) OR (alien) OR (travel) (Word variations have been searched)

#4 #1 AND #2 AND #3

**Web of Science**

TS=(tuberculosis OR "active tuberculosis" OR LTBI OR "latent tuberculosis infection") AND TS=(prevalence OR incidence OR "detection rate" OR epidemiology OR screening) AND TS=(migrant OR immigrant OR foreigner OR alien OR travel OR visa)
