# Supplementary material for: Prevalence of Tuberculosis among migrants under national screening programs: a systematic review and meta-analysis
Source: Glob Health Res Policy. 2025 Jun 23;10:24. doi: 10.1186/s41256-025-00424-y (PMC12183900; doi:10.1186/s41256-025-00424-y)
Supplement: Supplementary file 3 — Additional file 3. [file 41256_2025_424_MOESM3_ESM.docx]

Table S1 Meta-Analysis Data Extraction Form

| **Rank** | **Author** | **Year** | **Sample** | **TB cases** | **LTBI cases** | **Study period** | **Screening time point** | **Screening methods** | **Migrants group** | **Receiving country** | **Country of origin** | **STROBE rating** |
| --- | --- | --- | --- | --- | --- | --- | --- | --- | --- | --- | --- | --- |
| **1** |  |  |  |  |  |  |  |  |  |  |  |  |
| **2** |  |  |  |  |  |  |  |  |  |  |  |  |
| **3** |  |  |  |  |  |  |  |  |  |  |  |  |
| **·····** |  |  |  |  |  |  |  |  |  |  |  |  |

Table S2 Policy Information Extraction Form

| **Rank** | **Receiving Countries** | **Score** | **Income Level by World Bank** | **Incidence WHO estimated** | **Duration of Stay** | **Countries of Origin** | **Migrants Groups** | **Age** | **Active TBScreening Methods** | **Active TBScreening Time** | **LTBI Screening Methods** | **LTBI Screening Time** |
| --- | --- | --- | --- | --- | --- | --- | --- | --- | --- | --- | --- | --- |
| **1** |  |  |  |  |  |  |  |  |  |  |  |  |
| **2** |  |  |  |  |  |  |  |  |  |  |  |  |
| **3** |  |  |  |  |  |  |  |  |  |  |  |  |
| **·····** |  |  |  |  |  |  |  |  |  |  |  |  |
